# Extraction and Validation of Biomechanical Gait Parameters with Contactless FMCW Radar

**DOI:** 10.3390/s24134184

**Published:** 2024-06-27

**Authors:** Linyu Wang, Zhongfei Ni, Binke Huang

**Affiliations:** 1 School of Information and Communications Engineering, Xi’an Jiaotong University, Xi’an 710049, China; wliny99@stu.xjtu.edu.cn; 2 College of Physics and Electronic Engineering, Shanxi University, Taiyuan 030006, China; zhongfei_ni@sxu.edu.cn

**Keywords:** FMCW radar, gait parameters, reliability validation, Wilcoxon test, motion capture

## Abstract

A 77 GHz frequency-modulated continuous wave (FMCW) radar was utilized to extract biomechanical parameters for gait analysis in indoor scenarios. By preprocessing the collected raw radar data and eliminating environmental noise, a range–velocity–time (RVT) data cube encompassing the subjects’ information was derived. The strongest signals from the torso in the velocity and range dimensions and the enveloped signal from the toe in the velocity dimension were individually separated for the gait parameters extraction. Then, six gait parameters, including step time, stride time, step length, stride length, torso velocity, and toe velocity, were measured. In addition, the Qualisys system was concurrently utilized to measure the gait parameters of the subjects as the ground truth. The reliability of the parameters extracted by the radar was validated through the application of the Wilcoxon test, the intraclass correlation coefficient (ICC) value, and Bland–Altman plots. The average errors of the gait parameters in the time, range, and velocity dimensions were less than 0.004 s, 0.002 m, and 0.045 m/s, respectively. This non-contact radar modality promises to be employable for gait monitoring and analysis of the elderly at home.

## 1. Introduction

Gait activities, an indispensable part of human daily life, require intricate coordination between the nervous system and most musculoskeletal tissues, influenced by gender, age, occupation, and diseases [1]. Accurate measurement and analysis of gait parameters plays a vital role in assessing human health status, preventing potential risks, and formulating treatment plans. Specifically, it is applied in analyzing and diagnosing tremors caused by neurodegenerative diseases (such as Parkinson’s disease and cerebral palsy) [2,3], and it contributes to detecting fall risk among the elderly during daily life [4,5].

Currently, various devices are used in gait monitoring, which is classified into wearable and non-wearable technologies. Wearable devices such as speed sensors, acceleration sensors or highly reflective fluorescent markers are attached to the interested joints of subjects, to acquire their gait information [6,7]. High-accuracy measurement of the parameters is achieved by wearable devices, but the wearing of the device will interfere with the subject’s behavior, resulting in differences between experimentation and daily life. Non-wearable devices mainly extract the gait parameters of the subjects by GAITRite^®^ [8], WiFi [9], and radar [10], etc. GAITRite^®^ laid on the ground can measure with high accuracy the gait parameters of subjects, but it is expensive to deploy and difficult to monitor in a large area. Applying WiFi to measure gait parameters is applicable in multiple scenarios but with limited accuracy and sensitivity to the environment. Radar can not only achieve measurements in the range and velocity dimensions of the subjects in a large area through the radar echo signals, but it can also protect personal privacy and does not interfere with the daily activities of the subjects. Consequently, radar technology holds promising prospects for gait analysis in indoor scenarios.

With the wide application of radar, a subject’s motion features can be conveniently analyzed by micro-Doppler signatures. Millimeter-wave radar has wide application, including human identification [11], eye blink detection [12], and heartbeat prediction [13]. With the integration of deep learning, radar can also recognize the different behaviors of subjects, such as walking, running, and squatting [14], as well as different gestures [15]. A series of gait parameters were obtained by collecting the lower-limb data of subjects on a treadmill, using continuous wave (CW) radar, which was achieved by separating the Doppler frequency envelope of the toe, ankle, and knee [10]. Radar and Vicon or IMUs devices were simultaneously manipulated to collect gait data to verify the accuracy of gait parameters extracted by Doppler radar [16]. However, these experimentations were usually performed in restricted laboratory scenarios, such as subjects walking on a treadmill, or only the lower-limb data of subjects being collected instead of whole-gait data, which is not conducive to the monitoring and analysis of the gait features of a subject in daily-life scenarios, such as at home or in a working environment.

This work collected the subjects’ natural-gait data, using FMCW radar in a laboratory setting. Combined with the micro-Doppler feature of the subjects, the strongest signal from the torso in the velocity and range dimensions and the enveloped signal from the toe in the velocity dimension were separated. The gait parameters, such as step time, stride time, step length, stride length, torso velocity, and toe velocity, were extracted. Simultaneously, the subjects’ gait data were collected by the Qualisys system as the ground truth to validate the reliability of the gait parameters extracted by the radar.

The major contributions of this article can be summarized as follows:

1. FMCW radar was employed to collect the gait data of the subjects, followed by preprocessing the data into an RVT cube. Subsequently, the 2D OSCA–CFAR algorithm and dilation and erosion algorithms were implemented, to extract the subjects’ gait information. The torso signals were extracted both from VT and RT spectrograms, and the toe signal was extracted from a VT spectrogram. Moreover, the peak-velocity time points of the signals could be used to divide continuous steps, facilitating parameters’ extraction.
2. The reliability of the gait parameters extracted by the radar was verified with ground truth gait parameters, as measured by the Qualisys system. The Wilcoxon signed-rank test was applied, to determine whether the medians of the paired gait parameters from the same device but different directions were equivalent. The ICC was utilized to evaluate the consistency of paired gait parameters collected by two devices, and the difference values of the paired gait parameters between the total subjects were displayed as Bland–Altman plots.

The main structure of the article is organized as follows. In Section 2, we introduce the work of the data collection and preprocessing procedures. Section 3 describes the proposed method for extracting the gait parameters. Section 4 presents the experimental results and reliability validation, and, finally, the conclusion is presented in Section 5.

## 2. Data Collection and Preprocessing

### 2.1. Gait Data Collection

**Figure 1:**
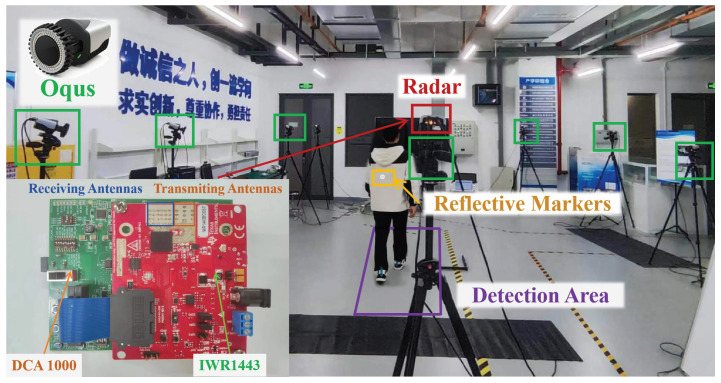
Experimental scenario with radar and Qualisys systems.

A Texas Instruments (TI) 77 GHz FMCW radar IWR1443 EVM [17] and a data-capture card DCA1000 EVM [18] were used to collect the raw gait data. The experimental scenario was in the No. 2 Building of Xi’an Jiaotong University iHarbour Campus, as shown in Figure 1, where the radar system was mounted on a tripod stand at a height of 2 m and placed 1 m away from a detection area with a size of 4 m × 1 m.

A three-dimensional (3D) motion capture system (eight high-speed infrared cameras, model Qqus, Qualisys, Sweden) was concurrently deployed to collect the gait data. The Qualisys system recorded body segment kinematics from several reflective markers at 100 Hz. The makers were attached to the interested body parts, including torso and toe.

The detailed radar configuration parameters are listed in Table 1. With this configuration, the FMCW radar can achieve a recording rate of 25 frames per second. Single input multiple output (SIMO) with one transmitting antenna and four receiving antennas, onboard-etched, was configured. The antenna peak gain was more than 10.5 dBi across the frequency band of 76 to 81 GHz. The beamwidth of the antenna design could be determined from the radiation patterns. For example, the horizontal and elevation 3 dB beamwidth were approximately ±28 and ±14 degrees at 78 GHz, respectively. Nine subjects (four males and five females, aged from 23 to 26 years old, weights from 50 to 75 kg, and of heights between 158 and 185 cm) walked forward and away 20 times in the line of sight (LOS) direction with radar.

### 2.2. Data Preprocesing

**Figure 2:**
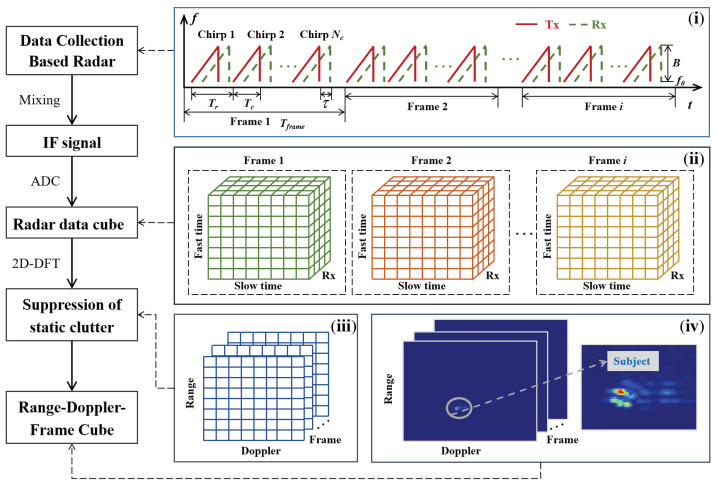
Flow chart for radar data preprocessing.

The flow chart for the radar data preprocessing with signal structure diagrams is detailed in Figure 2. First, the FMCW radar transmits chirp sequences frame by frame. The transmitting *m*th chirp signal is

(1) sTx,m(t)=cos2πf0t−mTr+St−mTr22=Reexpj2πf0t−mTr+jπSt−mTr2,mTr≤t<mTr+Tc,

where $m=1,2,3,\ldots ,N_{c}$ corresponds to the number of chirps in a frame, $f_{0}$ is the start frequency of the transmitted chirp signal, $T_{r}$ is the chirp repetition period, $S=B/T_{c}$ is the frequency slope, *B* is the sweep frequency bandwidth, $T_{c}$ is the chirp duration, and Re. represents the real part of the signal.

The transmitting signals were reflected by the subjects and obstacles from the detection area, then the radar received the delayed echo signals, which included the range and velocity information of the interested targets.

The intermediate frequency (IF) signal is obtained through mixing the receiving and transmitting signals, and then the raw data in discrete form could be represented as a matrix $x_{\mathrm{IF}}\left(n,m\right)$ by analog-to-digital converter (ADC) sampling:

(2) $$\begin{array}{c} x_{\mathrm{IF}}\left(n,m\right)=e^{j2\pi f_{\mathrm{IF}}\frac{n}{F_{s}}}{e^{j}}^{\varphi_{\mathrm{IF},m}}=e^{jn\omega_{\mathrm{IF}}}{e^{j}}^{\varphi_{\mathrm{IF},m}}. \end{array}$$

Here, $n=1,2,3,\ldots ,N_{s}$ corresponds to the number of ADC samples in a chirp, $F_{s}$ is the sampling frequency, $\omega_{\mathrm{IF}}=2\pi f_{\mathrm{IF}}/F_{s}$ is the discrete-time angular frequency, and $f_{\mathrm{IF}}=S\tau =\left(B/T_{c}\right)\tau =\left(2BR\right)/\left(cT_{c}\right)$, where *c* is the speed of light, $\varphi_{\mathrm{IF},m}=2\pi f_{0}\tau -\pi S\tau \left(\tau +2mT_{r}\right)$ is the phase of the IF signal, and τ=2R/c is the time delay between the receiving and transmitting signals.

As shown in Figure 2(ii), the raw radar data cube included fast-time sampling in each chirp along the vertical axis, slow-time sampling of multiple chirps in a frame along the horizontal axis, and four receiving channels in the third dimension.

The range profile for the subject is derived with discrete Fourier transform (DFT) processing in the fast-time dimension:

(3) $$\begin{array}{c} X_{\mathrm{IF}}\left(k,m\right)={e^{j}}^{\varphi_{\mathrm{IF},m}}e^{-j\frac{N_{s}-1}{2}\left(\frac{2\pi k}{N_{s}}-\omega_{\mathrm{IF}}\right)}\frac{\sin\left[\frac{N_{s}}{2}\left(\frac{2\pi k}{N_{s}}-\omega_{\mathrm{IF}}\right)\right]}{\sin\left(\frac{\pi k}{N_{s}}-\frac{\omega_{\mathrm{IF}}}{2}\right)},0\leq k<N_{s}. \end{array}$$

Based on the theory of range–DFT, the index *k* of $X_{\mathrm{IF}}\left(k,m\right)$ corresponds to the frequency $f_{k}=k\frac{F_{s}}{N_{s}}$, and it could be converted to the range unit with $r_{k}=f_{k}\frac{c}{2S}=k\frac{c}{2B}$ [19]. Thus, the range resolution is $R_{\mathrm{res}}=\frac{c}{2B}$ and the maximum range that could be detected by FMCW radar is $R_{\max}=\frac{F_{s}c}{2S}$.

Then, the DFT processing with Formula (3) in the slow-time dimension is represented as

(4) $$\begin{array}{c} Y_{\mathrm{IF}}\left(k,l\right)=X_{\mathrm{IF}}\left(k,0\right)e^{j\frac{N_{c}-1}{2}\left(\Delta \varphi -\omega_{l}\right)}{\left.\frac{\sin\left[\frac{N_{c}}{2}\left(\Delta \varphi -\omega \right)\right]}{\sin\left(\frac{\Delta \varphi -\omega }{2}\right)}\right|}_{\omega =\frac{2\pi l}{N_{c}}}, \end{array}$$

where $\Delta \varphi \approx 2\pi f_{0}\frac{2\Delta r}{c}=\frac{4\pi vT_{r}}{\lambda }$, $\lambda =c/f_{0}$, $T_{r}$ is the transmitted chirp interval and the target range changes $2\Delta r=vT_{r}$.

Based on the theory of Doppler–DFT, the index *l* of $Y_{\mathrm{IF}}\left(k,l\right)$ corresponds to the angular frequency $\omega_{l}=l\frac{2\pi }{N_{c}}$, and it could be converted to the velocity unit with $v_{l}=l\frac{\lambda }{2N_{c}T_{r}}$. Thus, the velocity resolution is $v_{\mathrm{res}}=\frac{\lambda }{2N_{c}T_{r}}$ and the maximum velocity that could be detected by the FMCW radar is $v_{\max}=\frac{\lambda }{4T_{r}}$.

According to the radar configuration parameters in Table 1, the range resolution and velocity resolution were 6.25 cm and 6.087 cm/s, respectively, and the maximum range and maximum velocity were 18.76 m and 6.09 m/s, respectively, which met the range and velocity testing requirements for human activities in a general indoor environment.

The radar data cube contained not only the radar echo information from the subjects, but also static clutter caused by stationary objects (such as walls, floors, etc.). Recognizing that the scattering of a stationary background is constant over time, the static clutter could be removed by average background subtraction [20], as shown in Figure 2(iii).

Thirdly, the range–Doppler spectrograms from the four receiving channels were incoherently summed, and the raw radar data wee processed frame by frame [11]. Figure 2(iv) shows the range–Doppler–frame data cube, which served as the source data for further gait parameter extraction, as discussed in Section 3.

## 3. Gait Parameters Extraction

In the range–Doppler–frame data cube derived from the preceding section, the relation between the Doppler frequency and the velocity of target is linear, which is

(5) $$\begin{array}{c} v=-\frac{f_{d}\cdot \lambda }{2}. \end{array}$$

Considering the consistency between frame and time, Doppler frequency and velocity, the range–Doppler–frame data cube could be converted to the range–velocity–time (RVT) data cube denoted as $\mathrm{RVT}\left(r,v,t\right)$, which was conveniently utilized to extract the gait parameters.

**Figure 3:**
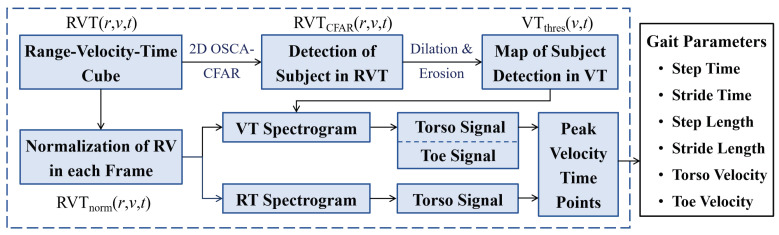
Flow chart of gait parameters extraction from FMCW radar RVT data.

Now the flow chart for the gait parameters extraction from the RVT data is shown in Figure 3, and the signal processing processes are depicted in detail as follows:

### 3.1. 2D OSCA–CFAR

A two-dimensional ordered-statistic cell-averaging constant false alarm rate (2D OSCA–CFAR) algorithm with an adaptive threshold was applied to the subjects’ information detection [21,22]. OS–CFAR was applied in the range dimension and CA–CFAR was applied in the velocity dimension. Specifically, for each sliding window of size $\left(2T_{r}+1\right)\times \left(2T_{v}+1\right)$ within the range–velocity (RV) spectrogram, the first step was to apply the method of OS–CFAR to the $2T_{r}+1$ velocity cells in the range direction, and the $k^{th}$ value was denoted as $X_{\left(k\right),v}$. The second step was to apply CA–CFAR to calculate the mean value of the $2T_{v}+1$ range cells of $X_{\left(k\right),v}$ in the velocity direction. The value $\mu \left(r,v\right)=\frac{1}{2T_{v}+1}\sum_{v=-T_{v}}^{T_{v}}X_{\left(k\right),v}$, the threshold factor α could depend on the threshold value, and the targets in each RV spectrogram could be separated as binary values:

(6) $$\begin{array}{c} RV_{\mathrm{CFAR}}\left(r,v\right)=\left\{\begin{array}{c} 1,\mathrm{RV}\left(r,v\right)\geq \alpha \cdot \mu \left(r,v\right)\\ 0,\mathrm{RV}\left(r,v\right)<\alpha \cdot \mu \left(r,v\right) \end{array}\right.. \end{array}$$

The $RV_{\mathrm{CFAR}}$ for each frame could be concatenated in the time dimension as $\mathrm{RV}T_{\mathrm{CFAR}}$.

### 3.2. Velocity–Time (VT) Spectrogram and Range–Time (RT) Spectrogram

Normalization of the RV spectrogram in the time dimension could reduce the impact caused by different ranges from radar detection [23]:

(7) $$\begin{array}{c} RV_{\mathrm{norm}}\left(r,v\right)=\frac{\mathrm{RV}\left(r,v\right)}{\max\left\{\mathrm{RV}\left(r,v\right)\right\}}. \end{array}$$

**Figure 4:**
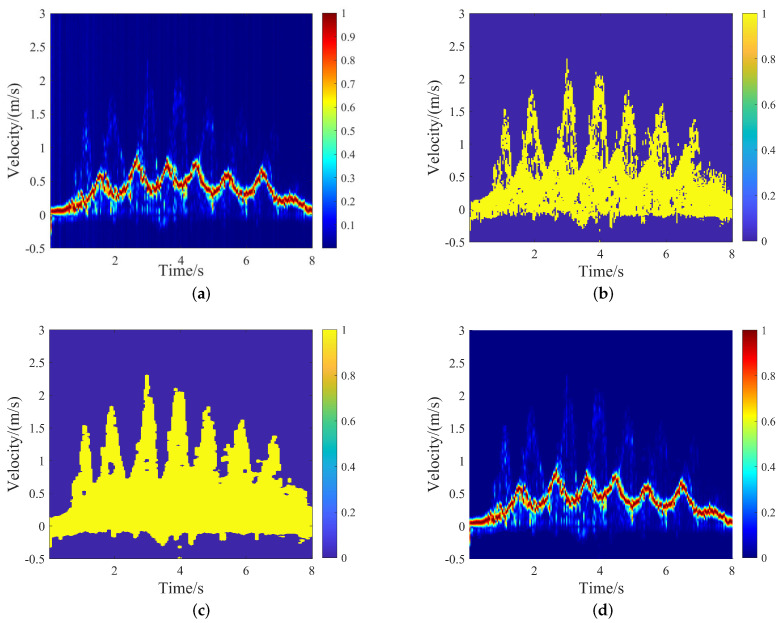
Extraction progress of subject’s VT spectrogram: (**a**) Normalized VT spectrogram. (**b**) VT spectrogram though CFAR processing. (**c**) VT spectrogram with dilatation and erosion processing. (**d**) VT spectrogram of the subject.

Concatenating the normalized spectrogram $RV_{\mathrm{norm}}\left(r,v\right)$ in the time dimension, the normalized three-dimensional radar data $\mathrm{RV}T_{\mathrm{norm}}\left(r,v,t\right)$ was achieved. Searching for the range cells with the strongest energy in the velocity direction obtained the VT spectrogram shown in Figure 4a as $VT_{\mathrm{norm}}\left(v,t\right)=\max_{r}\mathrm{RV}T_{\mathrm{norm}}\left(r,v,t\right)$. Similarly, we obtained the binary masker $VT_{\mathrm{CFAR}}\left(v,t\right)=\max_{r}\mathrm{RV}T_{\mathrm{CFAR}}\left(r,v,t\right)$ only including the velocity–time (VT) information of the subject, as shown in Figure 4b. Then, dilatation and erosion algorithms were employed for $VT_{\mathrm{CFAR}}\left(v,t\right)$ to fill in the small gaps and remove the small noise points, which was the binary plot $VT_{\mathrm{thres}}\left(v,t\right)$, as shown in Figure 4c, [10]:

The VT spectrogram in Figure 4d contains only the velocity information of the subject, which was realized by conveniently utilizing the Hadamard product operation for the normalized VT spectrogram $VT_{\mathrm{norm}}$ and the binary VT spectrogram $VT_{\mathrm{thres}}$, [24]:

(8) $$\begin{array}{c} \mathrm{VT}\left(v,t\right)=VT_{\mathrm{norm}}\left(v,t\right)⊙VT_{\mathrm{thres}}\left(v,t\right). \end{array}$$

**Figure 5:**
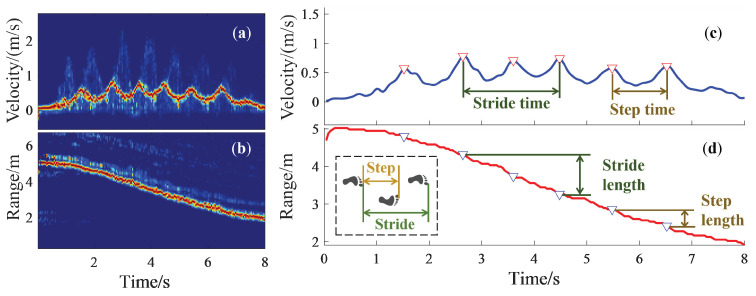
Torso signal extraction in VT and RT spectrograms: (**a**) The torso signal in the VT spectrogram. (**b**) The torso signal in the RT spectrogram. (**c**) Parameters extracted from torso velocity signal. (**d**) Parameters extracted from torso range signal.

The RT spectrogram was obtained in the normalized three-dimensional radar data $\mathrm{RV}T_{\mathrm{norm}}\left(r,v,t\right)$, just as the VT spectrogram depicted in Figure 5b:

(9) $$\begin{array}{c} \mathrm{RT}\left(r,t\right)=\max_{v}\left\{\mathrm{RV}T_{\mathrm{norm}}\left(r,v,t\right)\right\}. \end{array}$$

The range information from the torso signal and the signals caused by limbs and the rest of body of the subject are difficult to distinguish. However, the torso scattering energy is strongest in the range–time (RT) spectrogram, which is combined with the VT spectrogram in the gait parameters extraction.

### 3.3. Extraction of Gait Parameters

The strongest signals in the VT spectrogram and RT spectrogram are the reflection from the subject’s torso, and are depicted in red, as shown in Figure 5a and Figure 5b, respectively [25]. With the VT spectrogram shown in Figure 5a, the maximum-energy-extraction algorithm was used to separate the strongest energy points frame by frame, and the torso signal in the velocity dimension was obtained, as shown in Figure 5c by a blue solid line. In order to accurately detect the peak-velocity points, five-spot triple smoothing method was adopted, to get rid of tiny burrs [26].

The torso signal, which varied slowly and regularly with time, was extracted by the maximum-energy-extraction algorithm and depicted by a red solid line, as shown in Figure 5d.

When the foot touches the ground, the subject’s torso reaches peak velocity during walking [27]. By determining the moment at which the foot touches the ground, we can effectively extract peak-velocity points to separate each step from multiple gaits [28]. In addition, the absolute threshold of peak velocity was set to be at least 0.4 m/s, and the continuous peak interval was at least 0.44 s and no more than 1.5 s, which met the actual natural gait characteristics of almost all the subjects. The step time refers to the time interval between consecutive peak-velocity time points, and the stride time is the time interval between interval peak-velocity time points, as shown in Figure 5c. The first and last steps were usually adjustment steps, so these two steps did not factor in the subsequent gait parameters’ extraction.

Considering the consistency of VT spectrograms and RT spectrograms in time, the peak-velocity points in the velocity dimension could be projected into the range dimension. The step length refers to the range interval between consecutive peak-velocity points, and the stride length is the range interval between the interval peak-velocity points, which are shown in Figure 5d.

**Figure 6:**
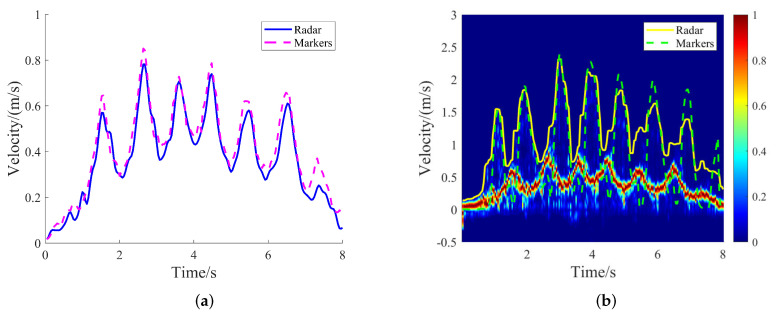
Comparison of torso and toe signals between radar and Qualisys: (**a**) Comparison of torso velocity signal. (**b**) Comparison of toe velocity signal.

The torso velocity is the value of the peak-velocity points on the torso signal. The torso velocity signals are shown in Figure 6a, and have good consistency with the signals measured by the FMCW radar and the Qualisys system. To achieve data synchronization in time, the generalized cross-correlation function was employed, to determine the time difference between them.

The toe velocity is the value of the peak-velocity points on the toe signal. The enveloped signal in the VT spectrogram is from the toes of the subjects, as shown by the yellow solid line in Figure 6b. We can see that the torso signal would occlusion the toes’ echo signal when the toes had smaller velocity than the torso. The markers on the toes were used to obtain the velocity of the toes with the Qualisys system. As shown in Figure 6b, good consistency was achieved between the radar and the Qualisys measurements in determining the maximum velocity of the subjects’ toes.

Regarding the method of using the Qualisys system to measure gait parameters, the step time, stride time, torso velocity, and toe velocity were similarly extracted by the above method, using radar. However, based on its working principle, the 3D coordinates of the torso and toe were obtained directly. The step length could be obtained by calculating the Euclidean distance between consecutive peak-velocity time points, and the stride length is the Euclidean distance between interval peak-velocity time points. With the gait parameters from the Qualisys system as the ground truth, the reliability and accuracy of the gait parameters from the radar will be discussed in detail in Section 4.

## 4. Experimental Results and Analysis

The gait data of the subjects were successfully collected by radar and the Qualisys system in Section 2. Six gait parameters were extracted using the above method in Section 3, and the total number of samples for the measured gait parameters of every subject was not exactly the same. Accordingly, 40 individual samples for each gait parameter were randomly selected for comparison, to assess the consistency across different walking directions and measurement devices. To validate this consistency, the estimated mean and the standard deviation (SD), along with the Wilcoxon test, were employed. Furthermore, the intraclass correlation coefficient (ICC) and Bland–Altman (BA) plots were utilized, to evaluate the accuracy of the gait parameters measured by the two different devices.

**Figure 7:**
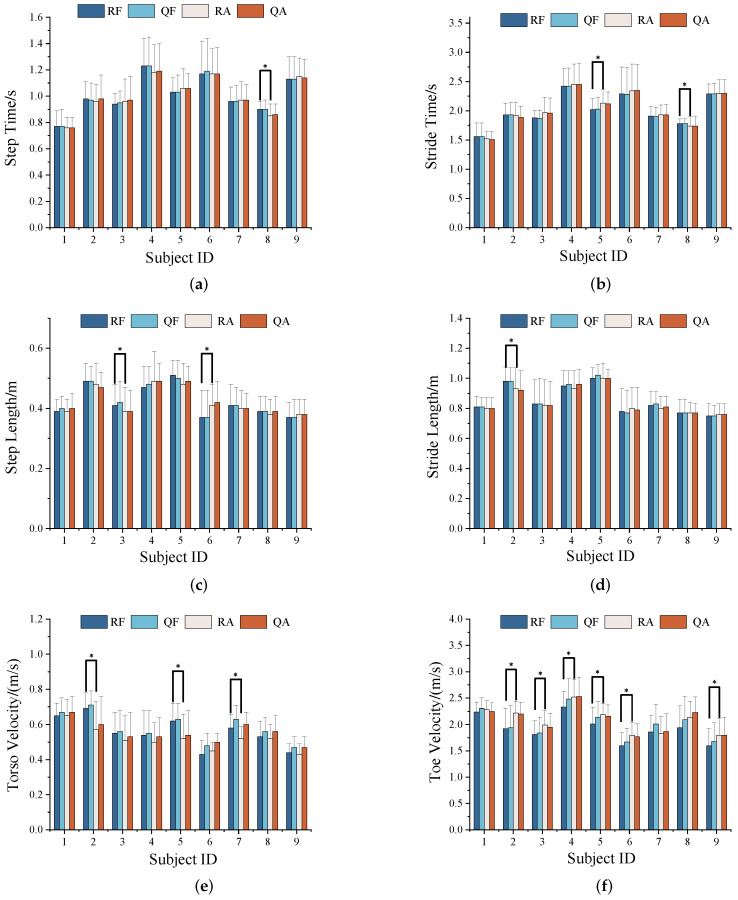
The mean, standard deviation, and Wilcoxon test of six extracted parameters: (**a**) Step time. (**b**) Stride time. (**c**) Step length. (**d**) Stride length. (**e**) Torso velocity. (**f**) Toe velocity (* represents rejections of the null hypotheses).

Figure 7a–f represent the mean and standard deviation (SD) of six gait parameters for nine subjects in bar charts, and they are specifically divided into radar for forward walking (RF), Qualisys for forward walking (QF), radar for walking away (RA), and Qualisys for walking away (QA), in different colors, respectively. The mean and SD values of the measured gait parameters in varying directions and devices were found to be insignificant for each subject, whereas the differences between individuals are obvious in Figure 7.

The Wilcoxon signed-rank test is used to measure the differences in the medians of the gait samples from the whole of the subjects walking in different directions [29]. The null hypothesis of the Wilcoxon test was $H_{\mathrm{RF}}=H_{\mathrm{RA}}$ and the confidence interval was set as 95%. The result of rejecting the null hypothesis meant that the differences between parameter samples walking in different directions was remarkable, and these are represented by symbol * in the bar charts, as shown in Figure 7. Further analysis revealed that the results of rejecting the null hypothesis of the gait parameters in the time and range dimensions were applicable to, at most, two subjects. For example, only Subject 02 rejected the null hypothesis in the judgment of stride length in Figure 7d. Nevertheless, there were more judgments rejecting the null hypothesis for toe velocity compared to the other parameters. This was likely due to the difficulty of radar signals fully covering the toes during reverse walking, resulting in partial signal loss and affecting the accuracy of the toe velocity measurements.

The agreement and accuracy of the individual gait parameters extracted using the FMCW radar compared to those measured using the Qualisys system were evaluated using the ICC. The ICC calculation values for all subjects across different directions are presented in Table 2. The ICC values of step time and stride time were generally greater than 0.92 and 0.97, respectively. The ICC values pertaining to step length and stride length were typically above 0.79. However, the ICC values of the gait parameters for back walking were no more than 0.90, which were smaller than those for forward walking. The significant difference was attributed to the paired–parameters samples from one of the subjects, potentially stemming from variations in sample selection. Moreover, the ICC values associated with the gait parameters in the velocity dimension typically exceed 0.96. As shown in Table 2, all the ICC values exceeded 0.79, which indicates good consistency between the two methods used for measuring gait parameters.

**Figure 8:**
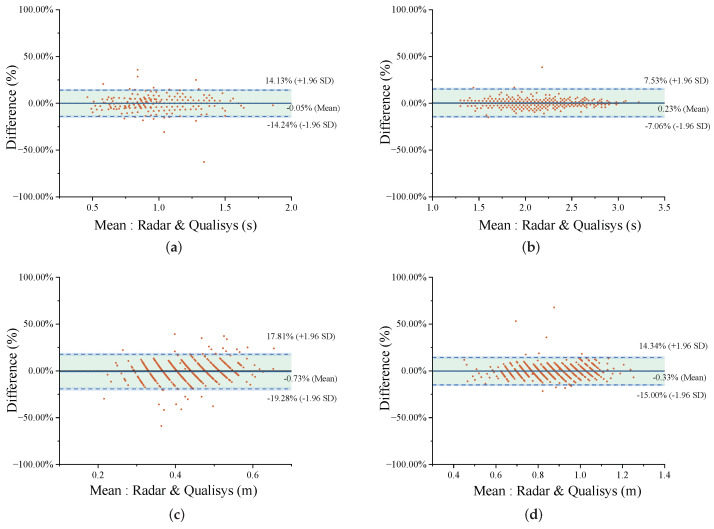

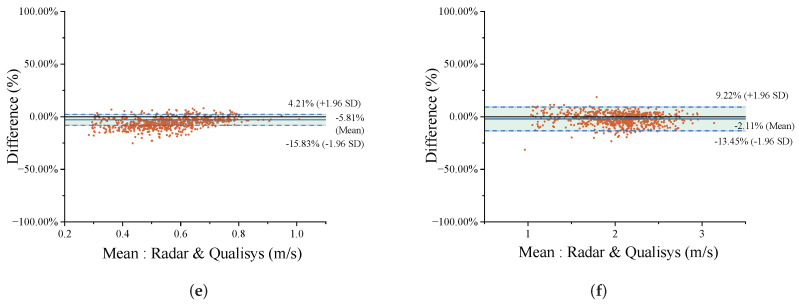
Bland–Altman plots of individual gait parameters using the FMCW radar and the Qualisys system: (**a**) Step time. (**b**) Stride time. (**c**) Step length. (**d**) Stride length. (**e**) Torso velocity. (**f**) Toe velocity.

Moreover, Bland–Altman plots are generated to graphically present the percentage differences between the paired gait parameters, and the values of the differences and the limits of agreements (LOAs) are evaluated between the FMCW radar and the Qualisys system measurements [30]. The Bland–Altman plots of individual gait parameters were shown in Figure 8. As shown in Figure 8a for step time, the absolute average of the percentage difference was 0.05%; this value corresponds to 0.001 s. In Figure 8b, for stride time the absolute average of the percentage difference was 0.23%, and the stride time error was 0.004 s. In Figure 8c,d, for step (stride) length, the absolute average of the percentage differences remained below 0.73%, specifically amounting to 0.002 m. In addition, the LOAs width of difference measurements in the range dimension exceeded those in the time and velocity dimensions. Moreover, the absolute average percentage differences in Figure 8e,f both remained below 5.81%, indicating a velocity error not exceeding 0.045 m/s.

## 5. Conclusions

For this paper, the biomechanical gait parameters of nine subjects were measured by FMCW radar and the Qualisys system. By processing the RVT data cube, torso signals in the velocity and range dimensions and enveloped signals in the velocity dimension were extracted. The peak-velocity time points were applied, to split continuous steps and to extract the proposed parameters, which are shown in bar charts within the mean and the SD. Moreover, the Qualisys system was synchronously utilized to measure the gait parameters of the subjects as the ground truth. The variations originating from various directions and across devices were examined through the application of the Wilcoxon test and the computation of the ICC, and the analysis of individual differences was facilitated by the utilization of Bland–Altman plots. The results indicate that the average errors of the gait parameters in the time dimension were no more than 0.004 s, that the average errors of the gait parameters in the range dimension were no more than 0.002 m, and that the average errors of the gait parameters in the velocity dimension were no more than 0.045 m/s. In conclusion, FMCW radar provides a non–cooperative and highly reliable method of measuring human gait parameters in indoor environments.

In our gait data collection, the walking subject was limited in the line of sight (LOS) direction with single input multiple output (SIMO) antenna. Considering the angle dependence of micro-Doppler signatures when the subject walks in a non-line of sight (NLOS) with radar, the multiple input multiple output (MIMO) configuration radar will be further exploited to track the subject location with angle information. Moreover, distributed radar can be deployed with signal fusion to improve accuracy and robustness in gait parameters extracting when the subject walks freely in indoor environments. In addition, only six typical spatial-temporal gait parameters were extracted by the proposed method; additional gait parameters reflecting subtle characteristics, such as swing/stance phase, single/double support time, step time variability analysis, etc., will be investigated in future work, which should be more indicative for early disease screening and clinical treatment evaluation.

## Figures and Tables

**Table 1:** Radar Configuration Parameters.

Parameters	Value
Start frequency (GHz)	77
Frequency slope (MHz/μs)	39.982
Chirp bandwidth (GHz)	2.398
Chirp duration (μs)	100
Chirp repetition period (μs)	160
ADC sampling rate (ksps)	10,000
Frame periodicity (ms)	40
Number of samples per chirp	360
Number of chirps per frame	200
Tx/Rx channels	1/4

**Table 2:** The ICC values for the gait parameters of the whole subjects.

Gait Parameters	ICC-F	ICC-A
Step Time	0.960	0.925
Stride Time	0.987	0.972
Step Length	0.910	0.792
Stride Length	0.938	0.867
Torso Velocity	0.980	0.975
Toe Velocity	0.962	0.961

ICC-F, ICC of parameters during forward walking; ICC-A, ICC of parameters during away walking.

## Data Availability

Data are available on request from the authors.

## Abbreviations

The following abbreviations are used in this manuscript:

ADC	analog-to-digital converter
CFAR	constant false alarm rate
DFT	discrete Fourier transform
FMCW	frequency-modulated continuous wave
ICC	intraclass correlation coefficient
MIMO	multiple input multiple output
RVT	range–velocity–time
SIMO	single input multiple output
